# “No End Point Detected”: A Unique Coagulation Profile Unmasking Dysfibrinogenemia

**DOI:** 10.1155/crh/8186265

**Published:** 2025-07-25

**Authors:** Kelsey Uminski, Xiu Yan Jiang

**Affiliations:** ^1^Division of Hematology and Hematologic Malignancies, University of Calgary, Calgary, Alberta, Canada; ^2^Alberta Precision Laboratories, Calgary, Alberta, Canada

## Abstract

Dysfibrinogenemia is a rare qualitative fibrinogen disorder that can present with bleeding, thrombosis, or both. We report a case of a young woman with first-trimester pregnancy loss and severe hemorrhage, whose coagulation tests reported “no end point detected” on PT, PTT, and Clauss fibrinogen assays. This pattern should prompt consideration of profound hypofibrinogenemia or qualitative fibrinogen defects. Fibrinogen replacement normalized PT and PTT and yielded measurable fibrinogen levels, enabling definitive diagnosis. Discordant activity and antigen levels, along with a pathogenic *FGB* variant, confirmed dysfibrinogenemia. This case underscores the importance of considering fibrinogen disorders in uninterpretable coagulation profiles and initiating early replacement.

## 1. Introduction

Dysfibrinogenemia is a rare coagulation disorder characterized by a qualitative fibrinogen defect that can result in bleeding, thrombosis, or both [1–3]. Diagnosis requires recognition of abnormal coagulation results, primarily the discordance between fibrinogen activity and antigen levels, and may include genetic testing [1–3]. This case highlights a patient with first-trimester miscarriage complicated by excessive vaginal bleeding, whose diagnosis was initially obscured by uninterpretable coagulation studies.

## 2. Case Presentation

A 30-year-old G1P0 female presented to medical attention with excess vaginal bleeding in the setting of retained products of conception (RPOC) at 12+ weeks gestation due to spontaneous abortion. Her pregnancy was conceived through in vitro fertilization and had been unremarkable until the week prior, when a nonviable intrauterine pregnancy was diagnosed and expectant management initiated. She had no known medical comorbidities and was taking ASA 162 mg for preeclampsia prevention. She reported no personal history of thrombosis but endorsed a history of menorrhagia on further inquiry. She stated no family history of bleeding or thrombosis.

Initial labs showed a hemoglobin of 94 g/L (range 120–160 g/L), which dropped to 74 g/L during resuscitation. Renal and hepatic functions were normal at presentation. Coagulation studies were collected reporting “no end point detected” for PT/INR, PTT, and fibrinogen (as measured by Clauss fibrinogen assay). The patient initially received two units of fresh frozen plasma. Following hematology and special coagulation lab consultation, fibrinogen concentrate (RiaSTAP) 6 g (88 mg fibrinogen concentrate/kg of body weight) was administered intravenously. Repeat coagulation studies following blood product receipt demonstrated an INR of 1.0 (range 0.8–1.2), PTT of 31 s (range 26–40 s), and fibrinogen of < 0.5 g/L (range 2.0–4.0 g/L). An additional 6 g of fibrinogen concentrate was administered. Repeat coagulation studies demonstrated normal INR and PTT, with fibrinogen of 6.2 g/L. The patient underwent a dilation and curettage with good hemostatic outcomes. She received tranexamic acid from the time of her presentation and continuing for 72 h postdilation and curettage.

The patient was subsequently followed up on discharge. Testing demonstrated fibrinogen < 0.5 g/L (Clauss fibrinogen assay) with a fibrinogen antigen level of 3.0 g/L [fibrinogen LIA (latex immunoassay) assay; range 1.6–4.3 g/L]. PT/INR and PTT remained normal. Genetic testing confirmed the presence of a heterozygous likely pathogenic variant in *FGB*(c.586C > T, p.Arg196Cys), establishing the diagnosis of dysfibrinogenemia. This specific variant (Fibrinogen Longmont) has been reported in the ClinVar database (Accession: VCV000016391.6) [4] and is associated with congenital dysfibrinogenemia. It has been identified in individuals with bleeding diatheses and is considered likely pathogenic [5–8], where in vitro studies suggest that this variant disrupts fibrinogen polymerization, leading to impaired clot formation [5].

## 3. Discussion

This case illustrates the diagnostic challenge of severely reduced fibrinogen, where PT/INR, PTT, and Clauss fibrinogen assays failed to generate a clot, leading to uninterpretable results. In our institution, testing was performed on the ACL TOP hemostasis analyzer (Werfen), which detects clot formation through a photo-optical turbidimetric method, which involves detecting changes in the turbidity of a sample as a clot form [9]. The reported “no end point detected” indicates that the analyzer was unable to detect clot formation within the test window—a phenomenon that may also be reported as “no clot detected” on other laboratory platforms. These interpretations reflect the analyzer's inability to generate a measurable clot-based result when fibrinogen was both critically low and functionally abnormal.

The fibrinogen Longmont variant [4], present in this patient, is known to cause assay-dependent discrepancies in clotting test results [10]. Optical analyzers relying on light-scatter techniques may fail to detect clot formation due to impaired fibrin polymerization, resulting in a “no clot detected” reading. Conversely, mechanical analyzers, which measure plasma viscosity changes, may successfully detect clot endpoints. This distinction, documented in a previous fibrinogen Longmont report, highlights the necessity of interpreting such uninterpretable results within the context of the assay method employed [10]. However, it is worth noting that this optical discrepancy is not consistently observed across all cases [11]; indeed, our patient demonstrated measurable PT and PTT results using optical detection methods upon repeat testing performed outside of the acute clinical episode. This underscores that assay performance may vary with clinical context and timing. At the time of this case, alternative clot detection methods such as mechanical endpoint analyzers or viscoelastic testing (TEG or ROTEM) were not available at our institution. Such alternative methods may help to confirm clot formation despite impaired polymerization affecting optical detection.

When PT/INR, PTT, or Clauss fibrinogen assays report “no end point detected,” it is crucial to interpret this result carefully, considering a broad differential that includes clinical factors, sample collection issues, and analytical variables. This includes critical hypofibrinogenemia, dysfibrinogenemia, and multifactor deficiencies as seen in disseminated intravascular coagulation or advanced liver disease [12–14]. The presence of anticoagulants—particularly heparin or direct thrombin inhibitors—may also interfere with clot-based assays and prevent endpoint detection [15]. Preanalytical factors such as gross sample hemolysis, icterus/hyperbilirubinemia, polycythemia as a result of a disproportionately high ratio of anticoagulant to plasma, or excessive anticoagulation relative to blood volume if the collection tube is not filled to capacity, as well as analytical issues including reagent or instrument malfunction, must also be ruled out [16–18]. In the acute care setting, profound hypofibrinogenemia from consumption or hemorrhage is a frequent etiology [19], but persistent abnormalities should prompt consideration of an underlying qualitative fibrinogen disorder.

PT/INR and PTT are global assays that measure the time to fibrin clot formation via the extrinsic and intrinsic pathways, respectively, and depend on the presence of functional fibrinogen [3, 20]. The Clauss fibrinogen assay quantifies functional fibrinogen by measuring the rate of fibrin polymerization in the presence of excess thrombin [1, 3, 12]. In cases of dysfibrinogenemia, particularly when the defect involves impaired cleavage by thrombin or polymerization of fibrin monomers, the Clauss method may report very low or unmeasurable values [1, 3, 12]. Although PT-derived fibrinogen was not measured in our patient, it is noteworthy that the fibrinogen Longmont variant often exhibits assay-dependent discrepancies in fibrinogen quantification. In particular, PT–derived fibrinogen results tend to be disproportionately low compared to those obtained with the Clauss thrombin clotting assay [21]. This phenomenon has been documented in dysfibrinogenemia cases and, when observed, may serve as an additional diagnostic clue to an underlying qualitative fibrinogen defect [21].

Some coagulation laboratories employ reflex testing protocols whereby failure of PT or PTT to reach an endpoint may automatically trigger a fibrinogen assay to evaluate for hypofibrinogenemia. However, in this case, the patient's underlying qualitative fibrinogen defect (dysfibrinogenemia), in combination with acute fibrinogen consumption due to hemorrhage and RPOC, rendered even this reflexive approach ineffective, as the Clauss assay itself failed to produce a measurable result.

Subsequent testing revealed a discordance between functional and antigenic fibrinogen levels, with a functional: antigen ratio < 0.7, which is diagnostic for dysfibrinogenemia [1, 2, 12]. Antigenic fibrinogen was measured using a fibrinogen LIA, an immunoturbidimetric method that quantifies total fibrinogen protein regardless of its functional capacity [3, 12]. The preserved antigen level alongside markedly reduced functional activity is a hallmark of qualitative fibrinogen defects [1, 3]. Although not performed in our patient, reptilase time has historically been considered useful in distinguishing dysfibrinogenemia from the effects of heparin or direct thrombin inhibitors, as it remains unaffected by thrombin inhibitors and is typically prolonged in dysfibrinogenemia [3]. However, current ISTH guidelines [3] indicate that reptilase time is not routinely recommended in the standard diagnostic work-up of congenital fibrinogen disorders due to limited sensitivity and specificity, and because definitive diagnosis relies primarily on functional and antigenic fibrinogen assays combined with genetic testing.

Fibrinogen was initially depleted due to acute consumption in the setting of RPOC and hemorrhage. RPOC has been associated with localized coagulation activation and fibrin deposition, leading to a low-grade disseminated intravascular coagulation-like state, exacerbating fibrinogen consumption [22]. Significant hemorrhage further accelerated depletion. This case underscores the importance of recognizing severe fibrinogen deficiency and qualitative defects as causes of uninterpretable coagulation studies, the utility of interpreting discordant Clauss and antigenic fibrinogen levels, and the need to promptly initiate fibrinogen replacement in obstetric hemorrhage, where fibrinogen plays a critical role in hemostasis. Identification of this congenital dysfibrinogenemia variant has significant implications for future pregnancy management. Even in the absence of prior bleeding history, women with dysfibrinogenemia face increased risks of miscarriage and obstetric hemorrhage [23]. Current guidelines recommend proactive fibrinogen concentrate replacement, aiming to maintain fibrinogen levels ≥ 1.0 g/L prior to conception and throughout pregnancy, and even higher (∼1.5 g/L) around the time of delivery, to mitigate these risks [23].

## Data Availability

Data are available within the article. Additional data are not available.
